# Is Neonatal Viremia a Possible Predictor of the Timing of Maternal Infection in Asymptomatic Congenital Cytomegalovirus Infection? A Retrospective Study

**DOI:** 10.3390/jpm15050165

**Published:** 2025-04-24

**Authors:** Fabio Natale, Giovanni Boscarino, Giuseppina Liuzzi, Fabrizia Bonci, Giuseppe Maria Albanese, Raffaella Cellitti, Antonella Giancotti, Francesco Franco, Barbara Caravale, Rosaria Turchetta, Ombretta Turriziani, Maria Giulia Conti, Gianluca Terrin

**Affiliations:** 1 Department of Maternal and Child Health, Policlinico Umberto I, Sapienza University of Rome, 00161 Rome, Italy; giovanni.boscarino@yahoo.com (G.B.); rafcell@hotmail.it (R.C.); antonella.giancotti@uniroma1.it (A.G.); mariagiulia.conti@uniroma1.it (M.G.C.); gianluca.terrin@uniroma1.it (G.T.); 2 National Institute for Infectious Diseases ‘Lazzaro Spallanzani’ (IRCCS), 00149 Rome, Italy; giuseppina.liuzzi@inmi.it; 3 Neonatology Unit, Sant’Eugenio Hospital, 00144 Rome, Italy; fabrizia.bonci@yahoo.it; 4 Department of Sense Organs, Sapienza University of Rome, 00161 Rome, Italy; giuseppemaria.albanese@uniroma1.it (G.M.A.); rosaria.turchetta@uniroma1.it (R.T.); 5 Lazio Regional Health Authority, 00145 Rome, Italy; frfranco@regione.lazio.it; 6 Department of Developmental and Social Psychology, Sapienza University of Rome, 00161 Rome, Italy; barbara.caravale@uniroma1.it; 7 Department of Molecular Medicine, Policlinico Umberto I, Sapienza University of Rome, 00161 Rome, Italy; ombretta.turriziani@uniroma1.it

**Keywords:** congenital cytomegalovirus infection, disease risk factor, risk predictor, asymptomatic infection, blood viral load

## Abstract

**Background:** Asymptomatic congenital cytomegalovirus (acCMV) infections represent 85–90% of all congenital CMV infection. The incidence of late-onset sequelae in these cases significantly contribute to the burden of CMV disease. The timing of maternal infection (TMI) has been identified as the main predictor of late-onset sequelae in acCMV infants, and follow-up programs in Europe are currently calibrated according to the TMI. Our aim was to evaluate neonatal viremia as a possible predictor of the TMI in acCMV infections. **Methods:** Plasma viral loads (PVLs) were assessed in the first month of life in a population of acCMV-infected newborns delivered by women who suffer a primary CMV infection during pregnancy. TMI was assigned to a trimester of pregnancy according to the maternal serological screening. PVLs were evaluated in relation to the TMI and gestational age (GA) at birth. **Results:** One hundred and ten newborns were, respectively, assigned to preconceptional (6.4%), 1st (27.3%), 2nd (38.2%), and 3rd (28.2%) trimester infections. Median neonatal PVLs values were significantly different between groups (*p* < 0.001). First-trimester infections exhibited significantly higher PVLs when compared with third-trimester ones (*p* < 0.001). Overall, PVLs showed an inverse correlation with GA at birth (*p* = 0.003). **Conclusions:** Median neonatal PVLs are significantly higher in 1st trimester infections if compared with 3rd trimester ones, but a wide overlap between PVL values prevent their possible use as a predictor of the TMI. In our population, a significant inverse relationship, mainly dependent on 1st and 2nd trimester infections, is demonstrated between PVLs and GA. Overall, fetal viremia is already decreasing weeks before the term of pregnancy.

## 1. Introduction

Congenital cytomegalovirus (cCMV) infections, usually classified as symptomatic and asymptomatic, are the leading viral cause of sensorineural hearing loss (SNHL) and neurodevelopmental impairment in children. Symptomatic infections, around 10–15% of all congenital infections, are burdened with a 30–60% incidence of neurological and audiological sequelae [1]. Infants with symptomatic cCMV infection demonstrate significantly higher baseline viremia when compared with asymptomatic infections [2].

Asymptomatic cCMV (acCMV) infections are the vast majority (85–90%). As compared with symptomatic ones, acCMV infections carry a lower risk (5–10%) of developing late-onset sequelae (mostly SNHL) but contribute significantly to the global burden of cCMV disease [1]. Efforts have been made to identify reliable predictors of late-onset sequelae in this population that may assist in the stratification of disease risk since birth and guide clinical decision making. For this purpose, several studies have investigated the potential of neonatal viremia as a predictor of late-onset sequelae in this population [2,3,4,5,6,7]. Some studies report a viral threshold associated with an increased risk of late-onset sequelae [2,3,4,5], while others suggest a threshold below which risk is absent [4,5,6].

Recent studies have clearly demonstrated that the incidence of late-onset sequelae in acCMV infection is almost exclusively related to 1st trimester infections [8,9,10]. As a result, a European consensus has recently recommended personalized follow-up procedures for acCMV infants according to the trimester of maternal infection [11]. The authors recommend prolonged audiologic and neurodevelopmental controls in case of 1st trimester infection and/or when the timing of maternal infection (TMI) is unknown; only standard pediatric care is recommended for 3rd trimester asymptomatic infections with normal hearing at birth [11]. Serologic screening to detect CMV primary infection in pregnancy is not a universal practice. Non-primary maternal infections have no clear-cut diagnostic criteria and cCMV neonatal infections may be undiagnosed [1]. For both these reasons, population-based screening programs have been proposed [12].

To date, the relationship between neonatal blood viral load at birth and TMI is unknown in neonates with acCMV infection. The primary aim of our study was to investigate this relationship by relating neonatal plasma viral loads (PVLs) with TMI in a population of infants with acCMV infection delivered by women who suffered a primary CMV infection during, or right before, pregnancy. The interplay between PVLs, TMI, and gestational age (GA) at birth was also investigated. This study aims to explore the potential of neonatal viremia as a predictor of the TMI in acCMV infections.

## 2. Materials and Methods

### 2.1. Study Population

This is a retrospective analysis of data collected in a cohort of cCMV-infected infants (inborn and referred), cared for at the Outpatient Service of Congenital–Perinatal Infectious Diseases of the Policlinico Umberto I, Rome, Italy, in the period January 2011–December 2019.

Inclusion criteria for the primary outcome were (1) an acCMV infection in infants delivered by women who suffered a primary CMV infection during pregnancy; (2) a CMV-PVL evaluation available within 30 days of life, if performed in our virology unit with the same assay; (3) the absence of any treatment with antiviral drugs during pregnancy.

Non-primary maternal infections (due to the current lack of assays able to provide an accurate TMI [1], and to a possible interference of preexisting IgG anti-CMV with viral replication), and cCMV symptomatic infections (unevenly distributed along all the trimesters of pregnancy [8], and known to be associated with higher blood viral load [2,4]) were not eligible for the study.

### 2.2. Timing of Maternal Infection and Gestational Age Determination

TMI was determined according to the results of maternal serological screening performed during pregnancy (IgG seroconversion, IgM, and IgG-avidity) and maternal signs/symptoms consistent with CMV infection. IgG avidity assays were interpreted according to manufacturers’ instructions. In cases in which trimester allocation was doubtful, additional criteria, as in Revello et al. [13], were applied. GA at birth for each infant was reported as completed weeks of pregnancy. An ultrasound determination of GA, when available, was utilized when the last menstrual period was uncertain.

Each maternal infection was assigned to a period of pregnancy (1st trimester: 0–13 weeks; 2nd trimester: 14–27 weeks; 3rd trimester: >27 weeks); preconceptional infections were considered those occurring in the 12 weeks preceding pregnancy.

### 2.3. Diagnosis of Neonatal Infection

cCMV infection was diagnosed by means of Polymerase Chain Reaction (PCR) assays (quantitative or qualitative) performed on urine or saliva within 3 weeks of life. When saliva tested positive, a confirmatory PCR test on urine was requested. The definition of symptomatic and asymptomatic infection has changed significantly over time, and some differences still persist between recently published consensus guidelines [14,15]. In our study, infected infants were evaluated for signs of cCMV infection, and were classified as asymptomatic when no clinical, instrumental, and laboratory abnormalities compatible with a cCMV symptomatic infection were detected. The diagnosis of a symptomatic infection was based on the presence of signs of central nervous system involvement (microcephaly, ventriculomegaly, cerebral calcifications, periventricular cysts, neuronal migration disorders, cerebellar hypoplasia, seizures, chorioretinitis, strabismus, optic atrophy, SNHL) pneumonia, persisting abnormality of liver function (elevated conjugated hyperbilirubinemia, elevated liver transaminases, hepato/splenomegaly), and persisting signs of bone-marrow suppression (thrombocytopenia, neutropenia, anemia). Other signs, the pathogenesis of which was not clearly attributable to cCMV infection (lenticulostriatal vasculopathy, germinal matrix cysts, isolated auditory neuropathy [16], isolated intrauterine growth restriction [IUGR], and isolated prematurity), were not considered signs of symptomatic infection.

### 2.4. Real-Time PCR Assay on Plasma

CMV-DNA was extracted from 250 µL of plasma samples collected in ethylenediaminetetraacetic acid (EDTA) using the Nuclisens EasyMag instrument according to the manufacturers’ instructions (Biomerieux S.p.A., Firenze, Italy). The extracted DNA sample was used for the CMV-DNA amplification by the commercially available TaqMan Real-Time PCR kit (ELITech Group S.p.A., Torino, Italy) on the ABI 7300 Real-Time PCR instrument (Applied Biosystem, Foster City, CA, USA). The PCR assay amplifies a specific sequence within the exon 4 region of the CMV MIEA (Major Immediate Early Antigen, HCMV *UL123*) gene. Due to patenting, the primer sequences are not available.

The analytical sensitivity of the assay, as a 95% or higher Limit of Detection (LoD) of the DNA amplification allows for the detection of the presence of about 10 genomic copies in 20 μL of DNA added to the amplification reaction, corresponded to 200 gEq/mL in specimens. The sensitivity of 95% was determined by a standard curve provided by the commercial TaqMan Real-Time PCR kit used in the present study.

PVL values are expressed as gEq/mL Log10 (conversion factors: 1 gEq/mL = 1 copy/mL = 0.3 IU/mL) The lower and upper limits of the linear measuring range were 250 gEq/mL (2.4 log10) and 25,000,000 gEq/mL (7.4 log10), respectively. The results, referred to as <250 or >25,000,000 gEq/mL, indicate that CMV DNA is detected but it is not accurately quantified. To aid in data analysis, values between the lower limit of quantification (LoQ) and the lower LoD (reported as <250 gEq/mL) and negative values have been arbitrarily replaced by values of 100 gEq/mL (2 log10) and 10 gEq/mL (1 log10), respectively. We decided to also include negative blood tests (with urine confirmation of cCMV infection) based on the possibility that fetal viremia might be short enough to be no longer detectable at birth.

Plasma samples had a processing time ≤ 72 h in the vast majority of cases (twice weekly PCR sessions in our virology unit). For the purposes of the statistical analysis, PVLs are expressed as gEq/mL log10 (log).

### 2.5. Standard Immunoglobulins Administration and Neonatal PVLs

Several CMV-infected mothers received standard intravenous human immunoglobulins (sIVIG) for prophylaxis of vertical transmission (sIVIG administration after diagnosis of CMV primary maternal infection) or possible therapy (sIVIG administration after diagnosis of fetal infection by means of amniocentesis). A relation between sIVIG administration and neonatal PVLs was investigated, in exposed and non-exposed infants, to exclude a possible bias in PVL values.

### 2.6. Statistical Analysis

Data analysis was performed using IBM Statistical Package for Social Science Statistics version 26.0 (SPSS Inc., Chicago, IL, USA). Normality was verified by Kolmogorov–Smirnov test. The median, interquartile range and minimum–maximum interval summarized the continuous variables, numbers and percentages the categorical variables. Comparison between groups was performed using the Chi^2^ test, and One-way Anova. A significance threshold of *p* ≤ 0.05 was used, except for the comparison of PVL log between different groups where a more conservative approach was utilized. In this case, a Bonferroni correction for multiple testing was applied, and only *p* values ≤ 0.0083 (0.05 ÷ 6) were considered statistically significant. The relation between gestational age and PVLs was verified by Spearman’s rank correlation coefficient. The sample size of the study was calculated on the basis of some preliminary data in our possession. To demonstrate a difference in blood viral load of at least 50% between infections contracted early in pregnancy (1st trimester) and those contracted later (3rd trimester), we calculated the need to enlist at least 30 infants (Power 95%, alpha error 0.01) with acCMV infection for each trimester of pregnancy.

## 3. Results

The primary outcome of our study was to investigate neonatal viremia as a possible predictor of the TMI in acCMV infections. Among 159 subjects with cCMV infection, 110 asymptomatic infants (1 twin pregnancy with both newborns infected) were eligible for the study [preconceptional infection: 7 infants (6.4%); 1st trimester: 30 infants (27.3%); 2nd trimester: 42 infants (38.2%); 3rd trimester: 31 infants (28.2%)]. Forty-nine/159 (30.8%) infants were excluded from the study due to the presence of one or more of the following causes: maternal infection not classified as primary or of an unknown nature, symptomatic congenital infection, the quantification of neonatal viremia performed at another hospital or beyond 30 days of life. None of the women underwent therapy with antiviral drugs during pregnancy.

The baseline characteristics of the study population in relation to the TMI are reported in Table 1.

Preconceptional infections are poorly represented in the sample size. Demographics do not show significant differences between groups.

### 3.1. Plasma Viral Load in Relation to the Trimester of Maternal Infection

Peripheral blood sampling for the determination of PVL was performed at a median age of 13.0 days of life (IQR: 8.5–18.5; min–max: 1–30). No significant difference in the time of blood sampling (possible source of bias when comparing PVL values) was observed between the four groups of maternal infection (Table 1).

**Figure 1:**
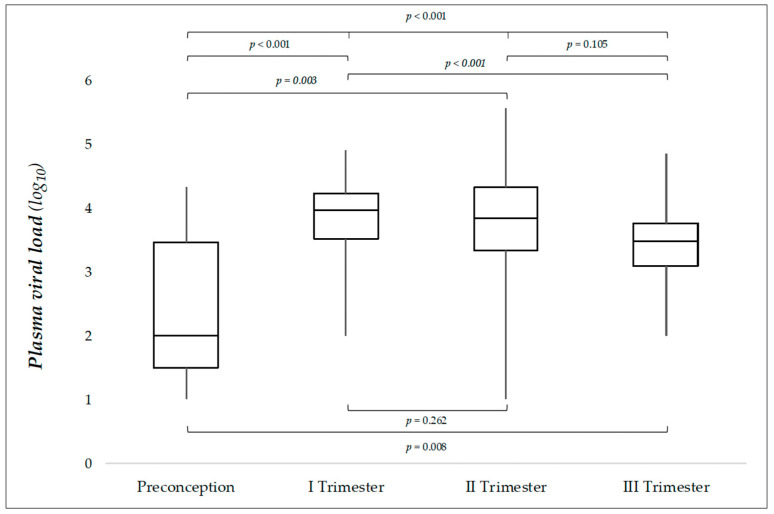
Distribution of plasma viral loads in relation to the trimester in which CMV maternal infection occurred (110 cases). Note: The box represents the interquartile range (IQR), with the line inside indicating the median. Whiskers extend to the minimum and maximum values. *p* values refer to comparisons between groups (One-way Anova). Values ≤ 0.0083 (after Bonferroni adjustment for multiple comparisons) are considered to indicate statistical significance.

Median PVL was 3.67 log (IQR: 3.27–4.18; min–max: 1–5.56) in the whole population, with significant differences across groups (*p* < 0.001) (Table 1). After Bonferroni adjustment for multiple testing, a *p* value ≤ 0.0083 (0.05 ÷ 6) was considered to indicate statistical significance. Third-trimester infections had significantly lower viral loads when compared with first-trimester infections (*p* < 0.001) (Figure 1). In detail, 3rd trimester median PVL value was 3.1 and 2.3-fold lower compared to those of 1st and 2nd trimester, respectively. Preconceptional infections exhibited PVLs significantly lower compared to each trimester of pregnancy (Figure 1). The distribution of PVLs across the four groups is illustrated by means of boxplots in Figure 1. Negative PVL values were 4/110 (3.6%) in our population, and 6/110 (5.4%) PVL values were under the LoQ of the assay.

After applying different PVL threshold (3 log, 3.5 log, 4 log), we failed to identify a clinically meaningful PVL threshold able to accurately predict the TMI. A PVL threshold = 4 log had a positive predictive value (PPV) of 82.3% in identifying 1st vs. 3rd trimester infections; the negative predictive value (NPV) was 64.4%. Diagnostic accuracy was even lower when comparing 1st vs. 2nd trimester infections: PPV and NPV were 46.7% and 61.9%, respectively.

### 3.2. Plasma Viral Load in Relation to Gestational Age at Birth

**Figure 2:**
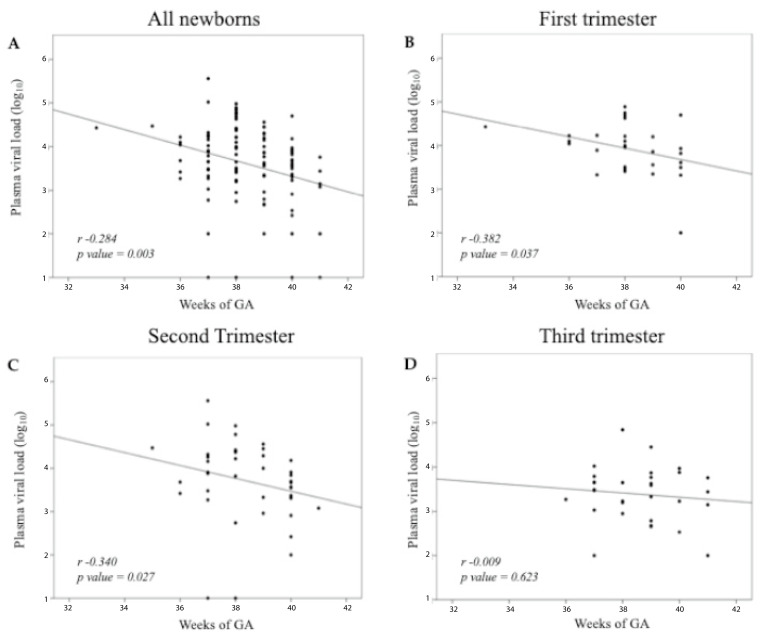
Distribution of neonatal PVLs in relation to gestational age at birth in the entire population (110 cases) (**A**) and in each trimester’s population (**B**–**D**). *r*: Spearman’s correlation coefficient rho.

A Spearman correlation was performed between GA at birth and PVLs for the entire population and for each trimester of maternal infection. Preconceptional infections, due to the small number, were included only in the analysis of the entire population (Figure 2A). Spearman’s correlation coefficient rho (*r_s_*) measures the monotonic relationship of the variables. A significant inverse relationship was detected in the global population (*r_s_* = −0.284; *p* = 0.003) (Figure 2A), indicating that lower gestational age at birth was associated with higher PVL. This relationship was mainly attributable to 1st (*r_s_* = −0.382; *p* = 0.037) (Figure 2B) and 2nd trimester (*r_s_* = −0.340; *p* = 0.027) (Figure 2C) maternal infections.

### 3.3. Neonatal PVLs in Relation to Maternal Immunoglobulin Administration During Pregnancy

Overall, 20/109 (18.3%) women received sIVIG (1–6 doses at 500 mg/kg/dose at monthly intervals). One twin pregnancy with both newborns infected did not receive sIVIG. These women received sIVIG as participants to a longitudinal prospective study on the prevention and/or therapy of fetal CMV infection [17].

A total of 8/30 (26.7%) infants with 1st trimester and 12/42 (28.6%) with 2nd trimester infection were exposed to sIVIG. No significant difference was detected in PVL between exposed and non-exposed infants belonging to the same trimester of maternal infection. (One-way Anova—1st trimester: *p* = 0.242; 2nd trimester: *p* = 0.764).

## 4. Discussion

Viremia at baseline has been repeatedly investigated as a possible predictor of late-onset sequelae in acCMV infections [2,3,4,5,6,7]. More recent studies highlight the predictive role of the TMI: Late-onset sequelae in acCMV infections are almost exclusively seen in 1st trimester maternal infections [8,9,10].

The primary aim of our study was to investigate the relationship between neonatal BVL values and TMI in a population of acCMV-infected newborns, in order to explore the possibility that neonatal viremia be able to predict the TMI.

In this study, we demonstrate that 1st trimester infections exhibit significantly higher PVLs at birth when compared with 3rd trimester and preconceptional infections. Preconceptional infections have significantly lower PVLs compared to all trimesters of maternal infection.

Preconceptional infections are poorly represented in our cohort (6.4%). The lower rate of vertical transmission, as compared with other TMI groups, may explain this result [10]. Though the small number prevent meaningful comparation with other groups, it should be noted that preconceptional infections collect the 40% (4/10) of all PVL values under the LoQ of our assay (including negative values), thus suggesting a lower viral replication, near the term of pregnancy, in this group. It is nevertheless worth underlining that a negative viremia at baseline, though associated with the absence of late-onset sequelae [4,6], does not accurately predict acCMV infections. In fact, up to 11% of cCMV symptomatic infections have non-detectable viremia at birth [18].

Our data show that the different distribution of maternal infections, from the preconceptional period along the three trimesters of pregnancy, may significantly affect the median PVL values in a population of acCMV infants. However, a wide overlap of the PVLs between the different trimesters of pregnancy prevented us from identifying a clinically meaningful PVL threshold able to predict the TMI. This should raise some concerns about the possibility that viral-load thresholds (as predictors of late-onset sequelae) could apply to a population different from the one in which the threshold is, a posteriori, calculated. A clinically relevant inter-assay variability in CMV-DNA results may further advise against the generalization of results from a single study [19]. Finally, due to possible selection bias, the results of retrospective studies are often not generalizable to the whole population [20].

Several studies have investigated CMV-DNAemia at birth in acCMV infections as a possible risk predictor for late-onset sequelae [2,3,4,5,6,7]. The results have been conflicting, with only some of these studies reporting a viral threshold above which the risk of late-onset sequelae is increased [2,3,4,5] or under which the risk is absent [4,6]. According to our results, a significant viral-load threshold in risk prediction could simply reflect the different viral-load values existing between earlier (known to be at higher risk of sequelae) and later (lower/no risk of sequelae) maternal infections.

A French multicentric study has recently analyzed neonatal CMV viral-load values in relation to the trimester of maternal infection [21]. The authors, similarly to what we observed in plasma, report significantly lower saliva viral load in third-trimester infections (when compared with earlier trimesters), but they did not find any significant difference in whole-blood viral loads. Though a significant difference in absolute values and clearance kinetics, possibly explaining our different results, has been reported in CMV viral-load monitoring using whole-blood vs. plasma assays in transplantology [22], a significant correlation between these two assays has been demonstrated when used in cCMV infection viral-load monitoring [23]. Further, the authors do not clearly identify neonates with asymptomatic infection and, unlike in our study, consider isolated IUGR as a sign of symptomatic infection. Finally, cases with blood viral load under the LoD are not included in the statistical analysis [21].

Blood viral loads in acCMV-infected infants start to decrease since birth to negativize at a median age of 3 months [5]. This study, to the best of our knowledge, is the first to investigate the kinetic of fetal CMV replication before birth. Our results on the interplay between GA and TMI demonstrate a significant inverse relationship in the entire population; PVLs are, on average, already decreasing well before the term of pregnancy. Of interest, this result is mainly due to first- and second-trimester infections, while in third-trimester infections, probably due to the shorter time elapsed from fetal infection to delivery, the PVL trend is less predictable.

The combined effect of TMI and GA at birth on neonatal PVL (higher PVL at birth and more rapid decline towards the end of pregnancy in the earlier maternal infections, and vice versa) suggests that viral replication in fetal peripheral blood peaks higher the earlier the fetal infection. Different PVL postnatal trajectories and, perhaps, times to negativity are to be expected for each trimester of maternal infection. According to our findings, an increasing PVL trend after birth could suggest a very recent fetal infection.

A possible explanation for our result resides in the interplay between the CMV intrinsic viral replication rate and the fetal immune system ontogenesis.

Both innate (Dendritic Cells, Natural Killer cells), and adaptive cellular immunity (CD4+ and CD8+ T cells) are involved in the control of CMV fetal infection [24,25], but their efficiency depends on the GA, affecting both quantity and quality of the fetal immune response, in which fetal infection occurs. Overall, data from the medical literature suggest a fetal immune response that starts to react to CMV infection from the second trimester of pregnancy, and progressively matures over time to complete in the first years of life [24,25,26,27,28,29].

Our results are consistent with such a view. Apart from the intrinsic viral replication rate, PVL at birth is the sum of two factors interacting with the developing fetal immune system. The first is the TMI. Earlier infections deal with a more immature immune system, and a less controlled viral replication will lead to higher PVL at birth (and vice versa). The second factor is represented by the time elapsed from fetal infection to birth, with longer times underpinning prolonged and progressively more mature immune responses able to curb the viral replication (and vice versa).

Our study has been carried out on one of the largest monocentric cohorts of acCMV-infected infants for whom a CMV-DNAemia was available in the neonatal period. All the plasma samples were prospectively analyzed in our virology unit with the same PCR assay. Of note, though PCR seems to be hardly influenced by prolonged storage [30], our samples had a processing time generally ≤ 3 days (twice a week PCR sessions) in agreement with harsher recommendations [31].

Little is known about the effect that preexisting anti-CMV maternal antibodies can exert on neonatal viremia at birth. In this light, it may be of interest to note that maternal sIVIG administration had no significant effect on neonatal CMV-DNAemia in our study, with this confirming the results of Revello et al. in which, unlike our population, anti-CMV hyperimmunoglobulins were administered [13].

Several limitations should be noted. Retrospective studies have multiple sources of bias [20]. Differences in baseline characteristics, selection bias, and recall bias are just some of the most frequently reported. However, the demographics did not show significant differences between groups in our study. Objective data (sex, weight, GA at birth) were easily retrieved from charts, and PVL values were all available in the database of our Virology Institute (no recall bias). Further, the assessment of the clinical/laboratory/instrumental screening for cCMV-infected newborns was up to only one clinician (FN) all along the study period (thus limiting the lack of homogeneity) [20].

Only primary maternal infections were eligible for the study but amniocentesis confirming the timing of fetal infection was available only for a small percentage (mostly belonging to the first trimester) of these infections. Though the TMI is commonly used as a surrogate for the timing of fetal infection, the possibility that fetal infection has occurred later than we estimated must be considered. According to a recent systematic review and meta-analysis [32], this possibility occurs in 8.0% (95% CI, 5.0–13.0%) of all cases.

Our data cannot apply to non-primary maternal infections. These infections represent about half of all cCMV infections in Europe [33], but are over 90% in low-income countries [34]. Currently available diagnostic assays do not allow for a precise timing of maternal infection in non-primary infections [1], and this led us to exclude these infections from the study. Nonetheless, the possibility that the timing of fetal infection could exert a significant effect on PVL at birth also in non-primary infections should be considered. Finally, we did not perform CMV genotyping on neonatal blood samples. Overall, studies investigating a possible association between different human CMV genotypes and cCMV disease have yielded contradictory results. A clear correlation between acCMV infection and certain genotypes has not been established [35]. However, the possibility that the intrinsic viral replication rate could vary in relation to different CMV genotypes cannot be excluded.

## 5. Conclusions

In a population of acCMV-infected infants, the median PVL at birth is significantly influenced by the distribution of the TMI in the population under observation. PVL values are significantly higher in 1st trimester infection when compared to 3rd trimester ones. Though, a wide overlap between PVL values in our population prevent the implementation of PVL as a reliable predictor of the TMI.

A significant inverse relationship exists between PVLs and GA. This relationship is mainly sustained by 1st and 2nd trimester maternal infections, in which PVLs show a declining trend in the last weeks before the term of pregnancy. As a whole, our results suggest higher peaks of viral replication in the fetus when the infection is contracted at an earlier stage during pregnancy.

Prospective studies in larger cohorts of acCMV-infected infants, possibly with determination of the time of fetal infection by amniocentesis, are needed to confirm our findings.

## Figures and Tables

**Table 1:** The baseline and virological characteristics of the enrolled population in relation to the timing of maternal infection.

Characteristic Cases (%)	Preconception 7 (6.4)	I Trimester 30 (27.3)	II Trimester 42 (38.2)	III Trimester 31 (28.2)	Total 110 (100)	*p* Value ^a^
Sex						
Female/Total	5/7	13/37	20/42	16/31	54/110	0.259 ^b^
(%)	(71.4)	(35.1)	(47.6)	(51.1)	(49.1)	
Birth weight (grams)						
Median	3190	3140	3215	3170	3200	
IQR	3020–3140	2525–3415	2900–3460	2980–3500	2870–3460	0.102 ^c^
Min–Max	2350–4280	1890–3980	2680–4180	2550–4210	1890–4280	
Gestational age (weeks)						
Median	39	38	38	39	38	
IQR	39–41	38–39	37–40	37–40	37–40	0.197 ^c^
Min–Max	37–41	33–40	35–41	37–41	33–41	
PVL sampling (days)						
Median	15.0	12.5	11.5	16.0	13.0	
IQR	10.0–22.0	6.0–17.0	6.0–17.0	11.0–21.0	8.5–18.5	0.394 ^b^
Min–Max	4.0–30.0	1.0–30.0	1.0–30.0	1.0–30.0	1.0-30.0	
PVL gEq/mL log10						
Median	2.00	3.97	3.83	3.47	3.67	<0.001 c *
IQR	1.00–4.15	3.51–4.22	3.32–4.32	3.03–3.77	3.27–4.17	
Min–Max	1.00–4.31	2.00–4.89	1.00–5.56	2.00–4.84	1.00–5.56	

Table legend: IQR (interquartile range); PVL (plasma viral load); ^a^: *p* value refers to comparation between groups; values ≤ 0.05 are considered to indicate statistical significance. ^b^: Chi-square test. ^c^: One–way Anova. *: values ≤ 0.0083 (after Bonferroni adjustment for multiple comparisons) are considered to indicate statistical significance.

## Data Availability

All data relevant to the study are included in the manuscript. Data will be available to any researcher from the corresponding author upon reasonable request.

## Abbreviations

The following abbreviations are used in this manuscript:

acCMV	Asymptomatic Congenital Cytomegalovirus
cCMV	Congenital cytomegalovirus
EDTA	Ethylenediaminetetraacetic Acid
GA	Gestational age
IQR	Interquartile Range
IUGR	Intrauterine Growth Restriction
LoD	Limit of Detection
LoQ	Limit of Quantification
NPP	Negative Predictive Value
PPV	Positive Predictive Value
PVLs	Plasma Viral Loads
PCR	Polymerase Chain Reaction
sIVIG	Standard Intravenous Immunoglobulins
SNHL	Sensorineural hearing loss
TMI	Timing of maternal infection
